# A Novel Immune and Stroma Related Prognostic Marker for Invasive Breast Cancer in Tumor Microenvironment: A TCGA Based Study

**DOI:** 10.3389/fendo.2021.774244

**Published:** 2021-11-18

**Authors:** Yizhou Huang, Lizhi Chen, Ziyi Tang, Yu Min, Wanli Yu, Gangyi Yang, Lili Zhang

**Affiliations:** ^1^ Department of Endocrinology, The Second Affiliated Hospital, Chongqing Medical University, Chongqing, China; ^2^ College of Pharmacy, Southwest Medical University, Luzhou, China; ^3^ Department of Rheumatology and Immunology, West China Hospital, Sichuan University, Chengdu, China; ^4^ Department of Breast and Thyroid Surgery, The Second Affiliated Hospital of Chongqing Medical University, Chongqing, China; ^5^ Department of Neurosurgery, The Second Affiliated Hospital, Chongqing Medical University, Chongqing, China

**Keywords:** breast cancer, tumor microenvironment, TCGA, tumor-infiltrating immune cells, ITK

## Abstract

**Background:**

Breast cancer (BC) is the most frequent cancer in women. The tumor microenvironment (TME), consisting of blood vessels, immune cells, fibroblasts, and extracellular matrix, plays a pivotal role in tumorigenesis and progression. Increasing evidence has emphasized the importance of TME, especially the immune components, in patients with BC. Nevertheless, we still lack a deep understanding of the correlation between tumor invasion and TME status.

**Methods:**

Transcriptome and clinical data were retrieved from The Cancer Genome Atlas (TCGA) database. ESTIMATE algorithm was applied for quantifying stromal and immune scores. Then we screened out the differentially expressed genes (DEGs) through the intersection analysis. Furthermore, the establishment of protein-protein interaction (PPI) network and univariate COX regression analysis were utilized to determine the core genes in DEGs. In addition, we also performed Gene Set Enrichment Analysis (GSEA) and CIBERSORT analysis to distinguish the function of crucial gene expression and the proportion of tumor-infiltrating immune cells (TICs), respectively.

**Results:**

A total of 1178 samples (112 normal samples and 1066 tumor samples) were extracted from TCGA for calculation, and 226 DEGs were obtained from this assessment. Further intersection analysis revealed eight key genes, including ITK, CD3E, CCL19, CD2, SH2D1A, CD5, SLAMF6, SPN, which were proven to correlate with BC status. Moreover, ITK was picked out for further study. The results illustrated that high expression of BC patients had a more prolonged overall survival (OS) time than ITK low expression BC patients (p = 0.009), and ITK expression also presented the statistical significance in age, TNM staging, tumor size classification, and metastasis classification. Additionally, GSEA and CIBERSORT analysis indicated that ITK expression had an association with immune activity in TME.

**Conclusion:**

ITK may be a potential indicator for prognosis prediction in patients with BC, and its biological behavior may promote our understanding of the molecular mechanism of tumor progression and targeted therapy.

## Introduction

Breast cancer (BC) is now becoming the most commonly diagnosed cancer in women worldwide, and it is estimated that approximately 2.3 million new cases (11.7%) have been diagnosed in 2020 (1). Although significant advances in treatments, including surgery, radiation and anti-cancer drugs, and diagnostics, have reduced cancer-related mortality, clinicians still face the challenge of therapy resistance-induced cancer recurrence, metastasis, and death (2, 3). The tumor microenvironment (TME) mainly consists of surrounding blood vessels, inflammatory cells, immune cells, lymphocytes, fibroblasts, and extracellular matrix (4). So far, increasing evidence indicates a strong association between TME and tumorigenesis, cancer cell proliferation, invasion, and metastasis (5). However, this connection also demonstrates a promising target that could be utilized for cancer therapy. Several studies have revealed immune cells in TME could influence tumor growth, which presents a hopeful treatment protocol concerning the tumor-infiltrating immune cell (TIC) in TME (6). For instance, tumor-infiltrating lymphocytes (TIL) have been reported to be a prognostic factor in patients with gastric cancer (7–9). High and/or intermediate immune scores suggest better disease‐free survival (DFS) and overall survival (OS) in patients with BC (10). Meanwhile, studies also illustrated that abnormal gene expression was significantly correlated with epigenetic alterations and expression signatures are predictive of clinical outcome (11, 12). Therefore, we extracted RNA-seq transcriptome data and clinical characteristics of BC from The Cancer Genome Atlas (TCGA) database and conducted the immune computational methods, such as ESTIMATE and CIBERSORT, to screen differentially expressed gene (DEG) which was related to immune component and clinical outcome. By combining the analysis of transcriptome and clinical data from TCGA, we established immune and stromal scores, which allowed us to screen precisely for prognostic DEGs. Thereafter, through the bioinformatics analyses, eight genes were considered key genes for BC patients, from which a novel gene, ITK, presented great predictive value in terms of the OS and clinical outcomes of BC patients. Further analyses demonstrated that ITK might become a new prognostic biomarker and therapeutic target in patients with BC.

## Materials and Methods

### Data Collection

Patients with invasive breast cancer that we had analyzed were retrieved from the TCGA database (https://portal.gdc.cancer.gov/). The data consists of two parts, including transcriptome profiling and the corresponding clinical characteristics (112 normal samples and 1066 tumor samples).

### ESTIMATE Algorithm for Immune Score, Stromal Score, and ESTIMATE Score

ESTIMATE algorithm was performed to assess stromal and immune microenvironment infiltration according to gene expression data through the R package “estimate” (https://www.r-project.org). Three kinds of scores, including Immune score, Stromal score, and ESTIMATE score, were shown to display a component ratio in TME.

### Survival Analysis and Clinical Relevance

Kaplan–Meier survival analysis was performed in Immune Score, Stromal Score, and ESTIMATE Score *via* R package “survival”. ESTIMATE Score consisted of both Immune Score and Stromal Score. The higher the scores, the larger number of components in TME. Six clinical characteristics were calculated for association with Immune Score, Stromal Score, and ESTIMATE Score respectively, including age, TNM staging, T classification (tumor size) of TNM stages, N classification (lymph node status) of TNM stages, M classification (distant metastasis) of TNM stages and histology. Wilcoxon rank-sum or Kruskal–Wallis rank-sum test was used for these comparisons, and *p* < 0.05 was considered significant.

### Classification in Stromal and Immune Groups and Identification of Differentially Expressed Genes

Tumor samples were divided into the high and low score groups according to the comparison to medians of stromal and immune scores. The R package “limma” was utilized to distinguish the DEGs. DEGs with |log2 FC| > 1 and false discovery rate (FDR) < 0.05 were considered significant. Meanwhile, DEGs that simultaneously upregulated or downregulated in stromal and in immune groups were identified validated. Visualization of DEGs was conducted by R packages “pheatmap” and “VennDiagram”.

### Functional Enrichment Analysis

Gene Ontology (GO) analysis and Kyoto Encyclopedia of Genes and Genomes (KEGG) pathway analysis with 226 DEGs were performed through R packages “clusterProfiler”, “enrichplot” and “ggplot2”. *p* and *q* < 0.05 were considered significant.

### Protein and Protein Interaction Network and COX Regression Analysis

PPI network was constructed in the Search Tool for the Retrieval of Interacting Genes database (STRING, 11.5 version) with a minimum required interaction score of 0.95. Reconstruction with 32 DEGs was managed by Cytoscape (version 3.7.2). In addition, we also performed univariate COX regression analysis using the R package “survival”. *p* < 0.05 was considered as the cut-off criteria.

### Connection Between ITK Expression and the Survival and Clinical Characteristics

The R package “survival” was used to compare low and high ITK expression, and “ggpubr” was applied to visualize the association between ITK expression and clinical characteristics. Wilcoxon rank-sum or Kruskal–Wallis rank-sum test was used for these comparisons, and *p* < 0.05 was considered significant.

### Gene Set Enrichment Analysis

GSEA was conducted by GSEA software (version 4.0.3) to pick out the functionally-relevant pathways regarding ITK expression. Nominal *p* < 0.01 were considered significant.

### Immune Cell Infiltration Calculation

CIBERSORT algorithm was utilized for evaluating the content of immune cells in tumor samples and visualized through barplot and heatmap. Expression proportion (high and low) and Pearson’s correlation analysis were performed in tumor samples. Shared immune cells were presented *via* the Venn diagram.

## Results

### Basic Information from TCGA Database and Process of Study

Transcriptome RNA-seq and clinical data were extracted from the TCGA database, including 112 normal samples and 1066 tumor samples. Clinical data consisted of six terms which were age (<=50 and >50), TNM staging (stage I, stage II, stage III, and stage IV), T classification of TNM stages (T1, T2, T3, and T4), N classification of TNM stages (N0, N1, N2 and N3), M classification of TNM stages (M0 and M1) and histology (infiltrating ductal carcinoma, infiltrating lobular carcinoma, medullary carcinoma, mucinous carcinoma, and others). The transcriptome RNA-seq data were analyzed by ESTIMATE and CIBERSORT algorithms. ESTIMATE algorithms were utilized to calculate immune and stromal scores, and CIBERSORT algorithms were used to calculate TICs proportion. DEGs obtained from intersection analysis of low and high Immune Score and Stromal Score groups were subsequently used to establish PPI network and perform the univariate COX regression analysis, following by the intersection analysis of core genes from the PPI network and top genes from COX regression analysis. Eight genes, ITK, CD3E, CCL19, CD2, SH2D1A, CD5, SLAMF6, and SPN, were obtained, and we then conducted further analysis, including survival and clinicopathological characteristics correlation analysis, GSEA, and correlation with TICs focused on ITK.

### Associations between the Scores and the Survival and Clinical Characteristics

**Figure 1:**
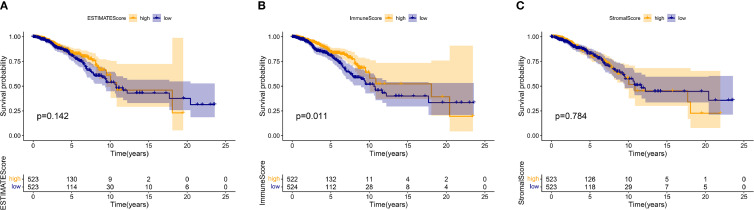
The relations between the survival and ESTIMATE Score, Immune Score, and Stromal Score in patients with BC. The Kaplan–Meier survival analysis was performed for ESTIMATE Score (*p* = 0.142), Immune Score (*p* = 0.011), and Stromal Score (*p* = 0.784), respectively. Comparisons were conducted in the high and low score groups and presented in orange and blue, respectively.

**Figure 2:**
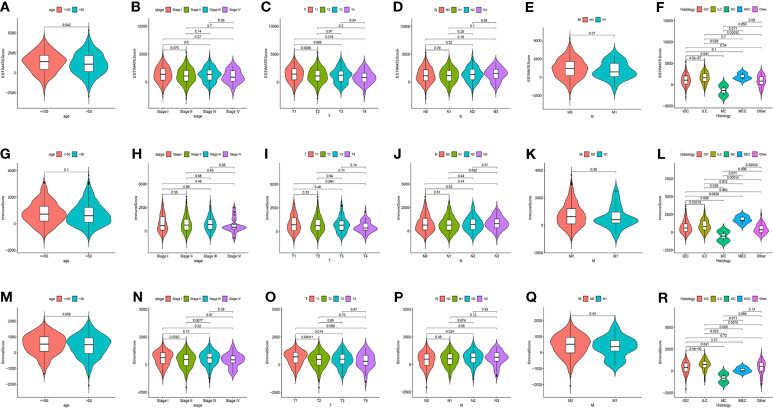
The relations between the Scores and Clinical Characteristics in patients with BC. **(A–F)** ESTIMATE Score in age, TNM staging, and histology. **(G–L)** Immune Score in age, TNM staging, and histology. **(M–R)** Stromal Score in age, TNM staging, and histology. IDC, Infiltrating Ductal Carcinoma; ILC, Infiltrating Lobular Carcinoma; MC, Mucinous Carcinoma; MEC, Medullary Carcinoma.

1046 tumor samples (20 samples with incomplete clinical data were excluded) were evaluated for the OS time and clinical relevance by ESTIMATE calculation methods and Kaplan–Meier survival analysis. ESTIMATE Score represents the sum of Immune Score and Stromal Score. In this analysis, patients with high Immune Score had a positive correlation with OS (*p* = 0.011) while Stromal Score and ESTIMATE Score did not reach the statistical significance (*p* = 0.784 and 0.142, respectively), which connote a positive role for immune components in patients with BC (**Figure 1**). Clinical data of patients with BC were also analyzed to determine whether TME had a relationship with clinicopathological characteristics (**Figure 2**). Our results illustrated that Immune Score was not associated with any clinicopathological characteristics except for histology, and scores of BC with different pathological types were significantly different. Stromal Score was negatively correlated with TNM staging of T classification (T1 vs. T2 and T3, *p* = 0.00011 and 0.014, respectively) (**Figure 2O**). In addition, ESTIMATE Score illustrated negative correlation with age (age ≤ 50 vs. >50, *p* = 0.042) and T classification (T1 vs. T2, T4, *p* = 0.0088 and 0.018, respectively) (**Figures 2A, C**). The results above signified a relationship between TME and BC progress or prognosis.

### Shared DEGs Were Mainly Enriched in Immune-Related Function

**Figure 3:**
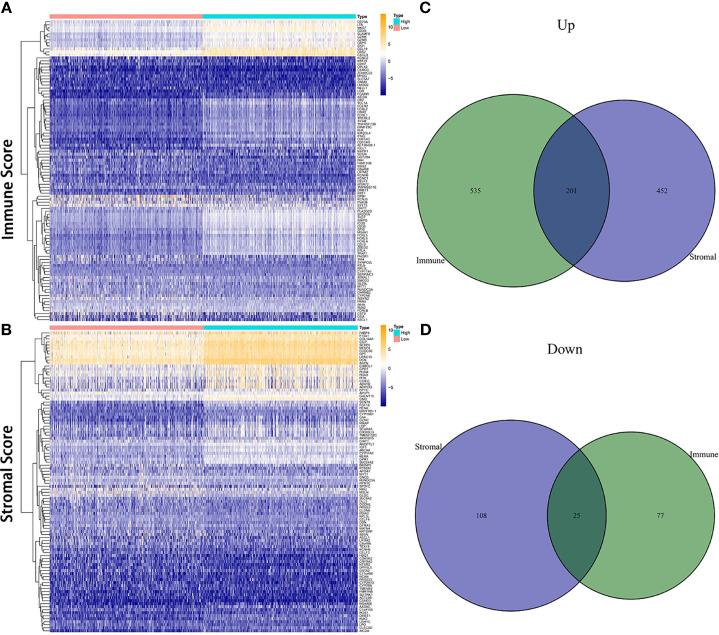
Heatmap and Venn diagrams for DEGs. **(A, B)** Heatmap for DEGs derived from comparing high score group and low score group in Immune Score and Stromal Score, respectively. Differentially expressed genes were determined by the Wilcoxon rank-sum test. **(C, D)** Venn diagrams for shared DEGs, including both upregulated and downregulated genes, in the Immune Score and Stromal Score groups.

**Figure 4:**
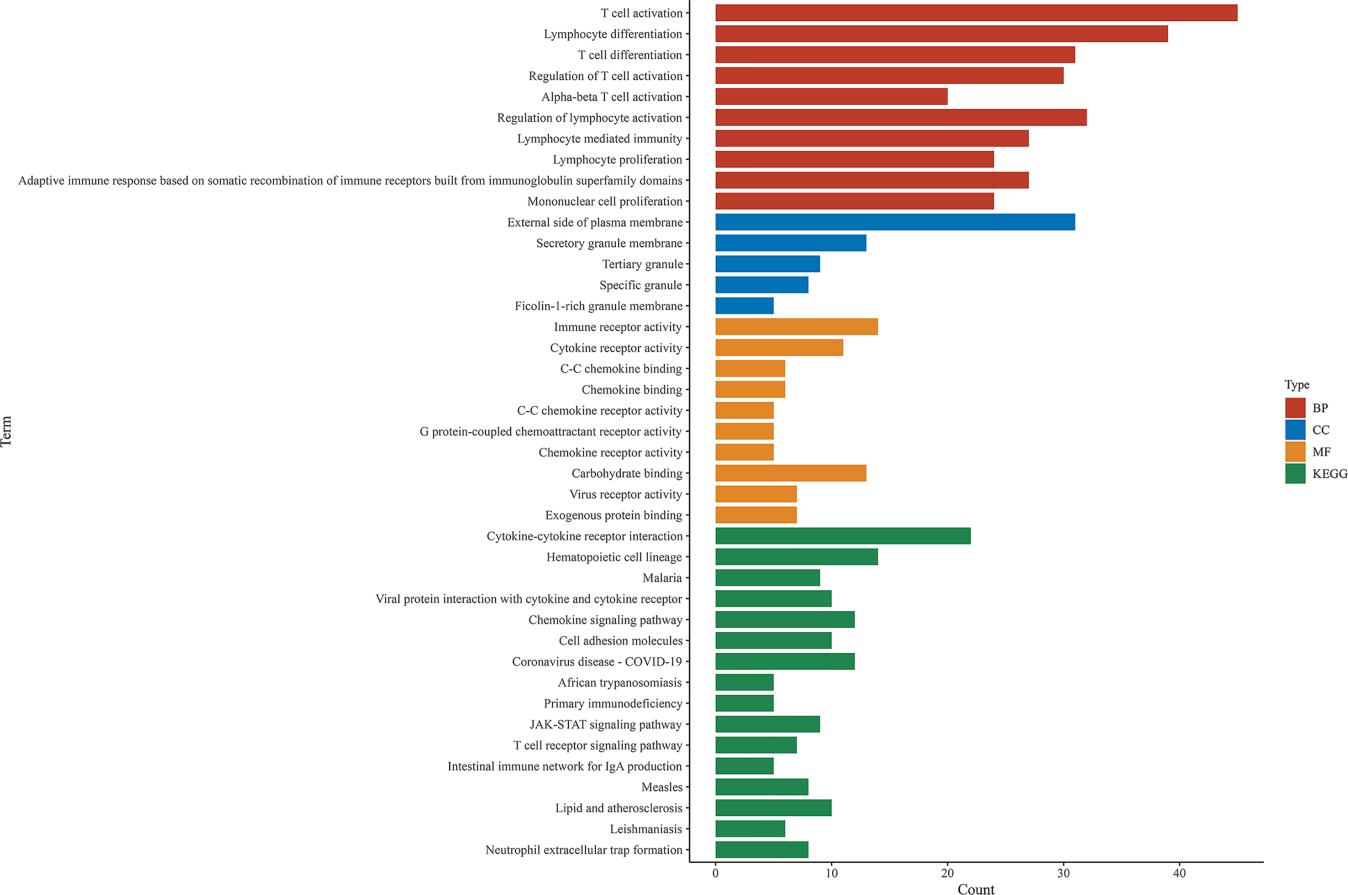
GO and KEGG enrichment analysis among shared DEGs. BP, biological process; CC, cellular component; MF, molecular function.

Comparison between the high and low scores in the immune and stromal groups was conducted to find a correlation between TME and gene expression. A total of 838 DEGs, 736 upregulated and 102 downregulated, were acquired from the immune group, while 786 DEGs, 653 upregulated, and 133 downregulated were obtained from the stromal group. The overlap of genes, including upregulation and downregulation, were considered as DEGs, in both immune and stromal groups. Accordingly, 201 upregulated genes and 25 downregulated genes were determined as final DEGs, which may be related to TME status (**Figure 3**). Besides, GO, and KEGG analyses were performed to explore the potential function among these genes. Results from GO and KEGG enrichment showed that the DEGs have a strong relationship with immune components (**Figure 4**). Consequently, DEGs seem to play an essential role in immune activity, which also suggests that immune factors may be a pivotal element in the TME of BC patients.

### Prognostic Genes Obtained *via* PPI Network and Univariate COX Regression

**Figure 5:**
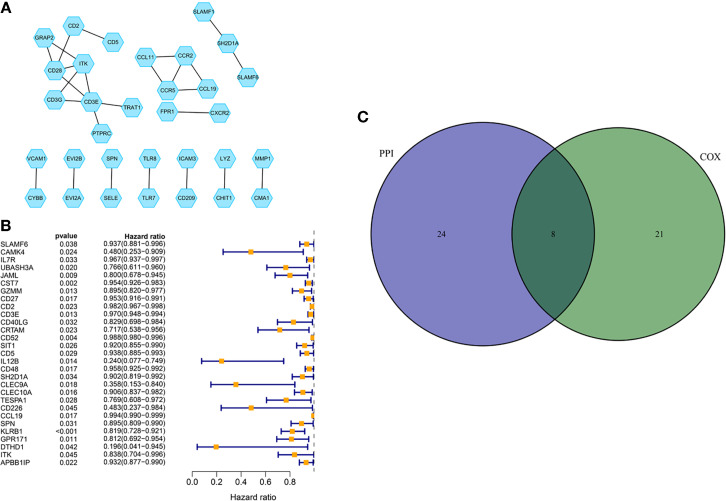
PPI network and COX regression analysis. **(A)** PPI network was established with a minimum required interaction score of 0.95 and visualized *via* Cytoscape. **(B)** Univariate COX regression analysis with the cut-off criteria of *p* < 0.05. **(C)** Venn diagram for the presenting of an intersection analysis between top 30 genes from the PPI network and the outcomes from univariate COX regression analysis.

A PPI network was established through the STRING database using 226 DEGs with a minimum confidence score of 0.95 and shown by Cytoscape software. Then top 30 genes were obtained by order of nodes counts (**Figure 5A**). To ensure the genes we extracted were key genes in BC prognosis, we also conducted the univariate COX regression analysis using 226 DEGs with a cut-off value of *p* < 0.05 (**Figure 5B**). Eventually, eight core genes, including ITK, CD3E, CCL19, CD2, SH2D1A, CD5, SLAMF6, and SPN, were acquired based on an intersection analysis between the top 30 genes from the PPI network and the outcomes from univariate COX regression analysis (**Figure 5C**). Due to the poor clinical relevance (CD3E, CD2, SH2D1A, CD5) and OS (CCL19, SLAMF6, and SPN), ITK was chosen for further analysis.

### Associations Between ITK Expression and the Survival and Clinical Characteristics

**Figure 6:**
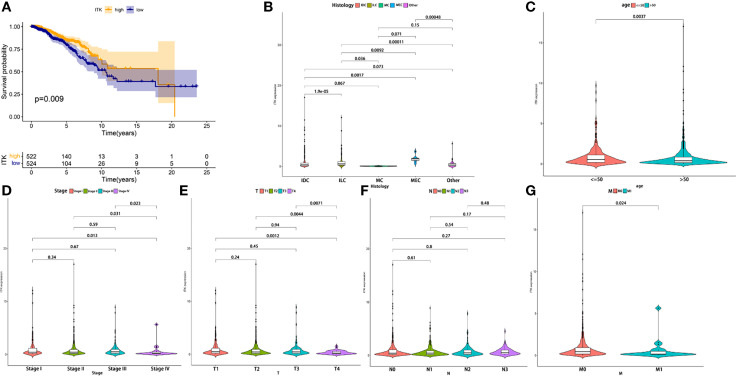
The correlation in the expression of ITK with the survival time and clinical characteristics. **(A)** The Kaplan–Meier survival analysis conducted for ITK expression. Comparisons were performed in the high expression and low expression groups and presented in orange and blue, respectively. **(B–G)** Comparison of ITK expression in histology, age, and TNM staging. IDC, Infiltrating Ductal Carcinoma; ILC, Infiltrating Lobular Carcinoma; MC, Mucinous Carcinoma; MEC, Medullary Carcinoma.

IL-2-inducible T-cell kinase (ITK) plays an important role in T cell signaling and producing various pro-inflammatory cytokines. In the present study, we divided all the samples into ITK high-expression group and low-expression group. The survival analysis demonstrated that ITK high-expression BC patients had a surprisingly longer OS time than ITK low-expression BC patients (*p* = 0.009), highlighting a positive connection between ITK expression and prognosis of patients with BC (**Figure 6A**). Meanwhile, ITK expression also presented statistical significance in age, TNM staging, tumor size classification, metastasis classification, and histology (**Figures 6B–G**). The ITK expression of young patients was higher than elderly patients (age ≤ 50 vs. >50, *p* = 0.0037). Patients in TNM Stage IV presented lower ITK expression than other stages (Stage IV vs. Stage I, Stage II, and Stage III, *p* = 0.013, 0.031, and 0.023, respectively). Patients with large tumor size (T4) expressed lower ITK (T4 vs. T1, T2, and T3, *p* = 0.0012, 0.0044, and 0.0071, respectively). Patients with metastasis represented higher ITK expression (M0 vs. M1, *p* = 0.024). The results further illustrated that higher expression of ITK was correlated with a better prognosis.

### ITK Related Biological Pathways

**Figure 7:**
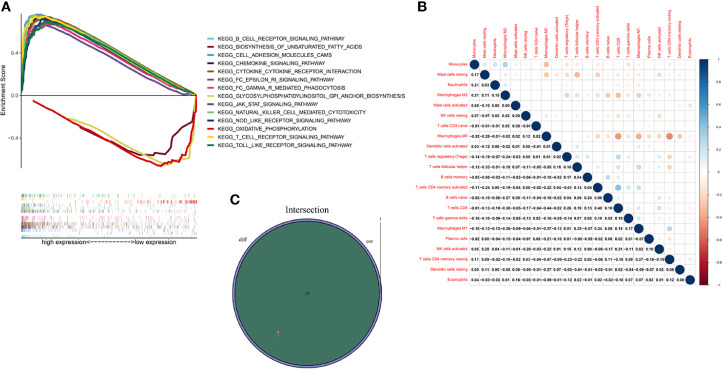
GSEA, TICs correlation and Venn diagram for ITK expression related TICs. **(A)** GSEA of BC samples in high and low expression groups. Nominal p < 0.01 were considered significant. **(B)** Heatmap was used for visualizing the correlation between 22 kinds of TICs. The deeper the color, the more significant the correlation is. Pearson coefficient was used for the significance test. **(C)** Venn diagram highlighted the ITK expression related immune cells, which were determined through difference and correlation analyses.

GSEA was performed to investigate the biological pathways associated with the expression of ITK. Our results demonstrated that high expression of ITK was mainly enriched in immune-related pathways, such as B cell receptor signaling pathway, cell adhesion molecules cams, and natural killed cell mediated cytotoxicity, which indicated an essential role of ITK in immune components of TME. In addition, the low ITK expression group was mainly involved in metabolism-related pathways, including oxidative phosphorylation, glycosylphosphatidylinositol GPI anchor biosynthesis, and biosynthesis of unsaturated fatty acids. (**Figure 7A**).

### Relationship Between ITK Expression and TIC

An analysis was managed to learn about the immune status in BC tumor samples utilizing CIBERSORT calculating methods. Twenty-two kinds of immune cells were obtained and exhibited in bar chart and heatmap, respectively (**Supplementary Figure 1** and **Figure 7B**). An intersection analysis was also implemented between difference and correlation analyses, and 15 kinds of immune cells turned out to be correlated with ITK expression, including nine positively related immune cells, such as T cells follicular helper, T cells gamma delta, T cells CD4 memory activated, T cells CD4 memory resting, T cells CD8, dendritic cells resting, macrophages M1, B cells naive and B cells memory, and six negatively related immune cells, such as mast cells resting, mast cells activated, macrophages M0, macrophages M2, NK cells resting and Neutrophils (**Supplementary Figures 2**, **3** and **Figure 7C**).

## Discussion

At present, research on TME has emerged as a hotspot due to its significance in treatments and prognosis (13–17). Without a doubt, TME in BC has also attracted the attention of lots of experts. In this study, we aimed to determine the TME-related genes correlated with tumorigenesis of patients with BC and may become a therapeutic target in clinical practice. A large number of studies have revealed the importance of the immune microenvironment in the progression of tumors. Therefore, we conducted the analyses to find out the related genes utilizing the transcriptome and clinical data from TCGA. Tumor purity, stromal and immune infiltration levels were assessed *via* ESTIMATE algorithm basing on transcriptome data. ESTIMATE Score, Immune Score, and Stromal Score were thus obtained from the calculation and used for the survival and clinical relevance analysis. A positive correlation between Immune Score and OS appeared to indicate that the immune component was a potential element for prognosis prediction, which was consistent with previous studies (18, 19). Thereafter, DEGs were acquired from intersection analysis, and enrichment analysis was performed using the TME-related DEGs. The results of GO and KEGG analyses illustrated that DEGs were mainly enriched in immune-related pathways. To further investigate the core genes, we established a PPI network and conducted the univariate COX regression analysis synchronously using DEGs. Finally, eight core genes, including ITK, CD3E, CCL19, CD2, SH2D1A, CD5, SLAMF6, SPN, were determined based on the above analyses. The correlation between CCL19, SLAMF6, and SPN and survival time was weaker than that of ITK (*p* = 0.011, 0.042, and 0.029). There is no statistical significance between CCL19, SLAMF6, SPN, CD3E, CD2, SH2D1A and CD5, and M classification of TNM (*p* = 0.066, 0.059, 0.11, 0.22, 0.11, 0.092 and 0.1, respectively). Therefore, CD3E, CCL19, CD2, SH2D1A, CD5, SLAMF6, and SPN were excluded for further analysis. Interestingly, CD2 was considered a prognostic biomarker in a previous study due to the strong correlation with OS. The possible reason for this contradiction may be that they did not consider the clinical factors (20). In addition, the BC samples were grouped into ITK high-expression and low-expression groups compared with the ITK median expression. The survival analysis showed that BC patients with ITK high expression had longer survival than that of ITK low expression. Further validation of GSEA and tumor-infiltrating immune cell proportion analysis supported that the levels of ITK affected the immune activity of TME and could be a prognostic biomarker for patients with BC.

ITK is a nonreceptor protein tyrosine kinase belonging to the Tec family, which is mainly expressed in T cells, NKT cells, and mast cells, and plays a significant role in T-cells signaling and cytokine production such as IL-2, IL-4, IL-5, and IL-13 (21–23). It has been assumed to participate in inflammation-mediated disease and hematological malignancies, such as allergic asthma, parasitic infection, and angioimmunoblastic T cell lymphoma (AITL) (24, 25); however, we still lack the evidence from the solid tumor. Although several novel ITK inhibitors were under development (BMS-509744, aminopyridone scaffold, thienopyrazole scaffold, CTA056, etc.), ibrutinib is still the most studied agent (23). Ibrutinib is a covalent small-molecule Bruton tyrosine kinase (BTK) inhibitor developed for B-cell malignancies. Surprisingly, it could also inhibit ITK and enhance the antitumor immune response to some extent (26, 27). There is credible evidence to confirm the good curative effect of ibrutinib in patients with malignant hematologic neoplasm, whereas it seems to have no significant effect in several solid tumors, including BC (28, 29), which is highly consistent with our result that higher ITK expression leads to longer OS time (*p* = 0.009). Similarly, patients with BC in stage IV, M1 classification, and T4 classification were discovered a low ITK expression and demonstrated the statistical significance (Stage IV *vs*. Stage I, Stage II and Stage III, *p* = 0.013, 0.031 and 0.023, respectively; T4 *vs*. T1, T2, and T3, *p* = 0.0012, 0.0044 and 0.0071, respectively; M0 *vs*. M1, *p* = 0.024). In addition, our result also indicated that older BC patients (age ≤ 50 *vs*. >50, *p* = 0.0037) expressed lower ITK, which was in accordance with an epidemiological investigation of US that 90% BC deaths occur in older age (age ≥ 50) (30). The results from our analysis based on data of BC patients confirmed that the immune components in TME contributed to the prognosis of patients. In particular, the expression of ITK significantly correlated with the invasion and metastasis of BC, which led to a poor prognosis.

Here we verified that ITK could be an indicator for BC patients due to the possible mechanism regarding TME. To investigate the relationship between ITK expression and TME in patients with BC, we, therefore conducted the GSEA. The analysis revealed that high ITK expression was mainly involved in immune-related signaling pathways such as T cell receptor signaling pathway, B cell receptor signaling pathway, Janus kinase/signal transducer and activator of transcription (JAK/STAT) signaling pathway, etc. Those results verified that ITK could modulate immune cells in BC *via* different pathways. Of note, low ITK expression enriched in metabolism-related pathways may imply a probability that the progression of BC was correlated with TME immune status. Thereafter, 22 kinds of immune cells were acquired from BC samples by CIBERSORT calculating methods.

Further intersection analysis exhibited 15 kinds of immune cells related to ITK expression, including nine positively related immune cells and six negatively related immune cells. Immune cells are initially a defense mechanism against cancers; however, the immune system could also promote the development of tumors in some cases. CD8+ lymphocyte is confirmed to be a tumor-suppressor in numerous cancers as well as a predictive factor for OS and DFS in BC (31, 32). Dendritic cells (DC) participate in the antitumor process due to their ability to cross-present antigens to CD4+ and CD8+ T cells for killing tumor cells. Likewise, the DC infiltration of tumor components means fewer metastases and a better clinical outcome (33). Tumor-associated macrophages (TAM) possess dominance in TME of BC. Generally speaking, macrophages are classified into M1 (classically activated) and M2 (type II alternatively activated). The M1 macrophages are regarded as antitumor macrophages due to their ability to release pro-inflammatory cytokines, reactive nitrogen, and oxygen intermediates (34). Conversely, the M2 macrophages play an important role in cancer promotion, tumor growth, angiogenesis, and metastasis, through the secretion of cytokines including IL4, IL-10, and transforming growth factor (TGF)-β (35, 36). Besides, several studies proved that patients with lower neutrophil-to-lymphocyte ratio (NLR) had a better prognosis than those with higher NLR (37, 38). Moreover, higher NLR led to increased mortality in patients with BC (39). The findings from other research above are consistent with our study, which emphasizes that ITK is a promising factor for predicting and treating patients with BC.

Our findings had provided novel insight for TME in tumor progression from different aspects utilizing bioinformatics and statistical methods. Rational use of ESTIMATE and CIBERSORT algorithms, followed by intersection analysis between PPI network and COX regression analysis, determined the TME related genes in BC. ITK was considered a potential biomarker for prognosis prediction through survival analysis and clinicopathological characteristics correlation analysis. In addition, our conclusion was further confirmed by GSEA and correlation analysis with TICs which also displayed the immune activity of ITK expression.

## Conclusion

In this study, multiple algorithms were utilized to display a relationship between tumorigenesis and the microenvironment infiltration in BC, and immune components were confirmed to significantly impact the progression of BC, which concretely reflected on the OS. Moreover, we picked out an immune-related gene, ITK, which demonstrated a strong clinical relevance and promising predictive power for patients with BC. Therefore, ITK has excellent potential to be a novel biomarker used for outcome prediction. Alternatively, the biological behavior of ITK depending on expression level and TIC phenotypes might provide new insights in targeted therapy and molecular biology progress.

## Data Availability Statement

The datasets presented in this study can be found in online repositories. The names of the repository/repositories and accession number(s) can be found in the article/**Supplementary Material**.

## Author Contributions

All authors contributed to the conception and design of the study. YH, LC, ZT, YM, and LZ organized the database. YH, LC, ZT, WY, and GY performed the statistical analysis. All authors wrote the first draft of the manuscript. All authors wrote sections of the manuscript. All authors contributed to the article and approved the submitted version.

## Conflict of Interest

The authors declare that the research was conducted in the absence of any commercial or financial relationships that could be construed as a potential conflict of interest.

## Publisher’s Note

All claims expressed in this article are solely those of the authors and do not necessarily represent those of their affiliated organizations, or those of the publisher, the editors and the reviewers. Any product that may be evaluated in this article, or claim that may be made by its manufacturer, is not guaranteed or endorsed by the publisher.
