# Supplementary material for: A Novel Immune and Stroma Related Prognostic Marker for Invasive Breast Cancer in Tumor Microenvironment: A TCGA Based Study
Source: Front Endocrinol (Lausanne). 2021 Nov 18;12:774244. doi: 10.3389/fendo.2021.774244 (PMC8636929; doi:10.3389/fendo.2021.774244)
Supplement: Supplementary file 1 [file DataSheet_1.docx]

**Supplementary Figure 1**


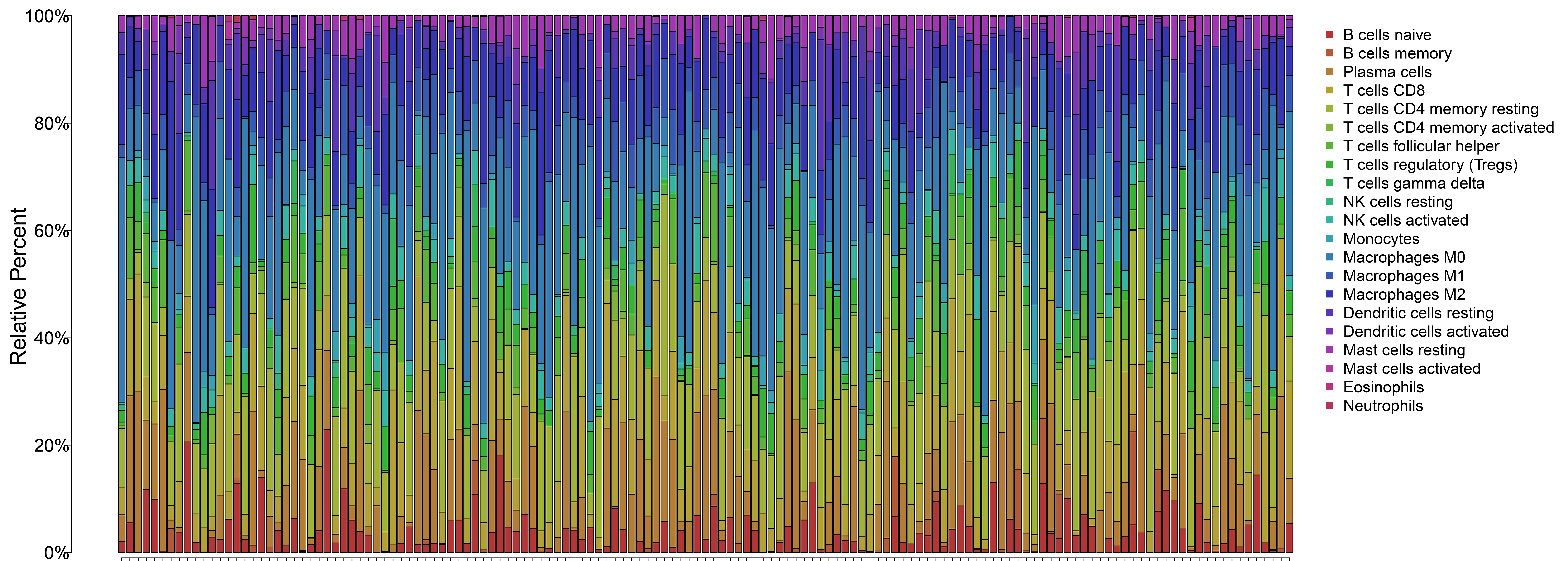


**Supplementary Figure 1** Proportion of 22 kinds of TICs in BC tumor samples.

**Supplementary Figure 2**


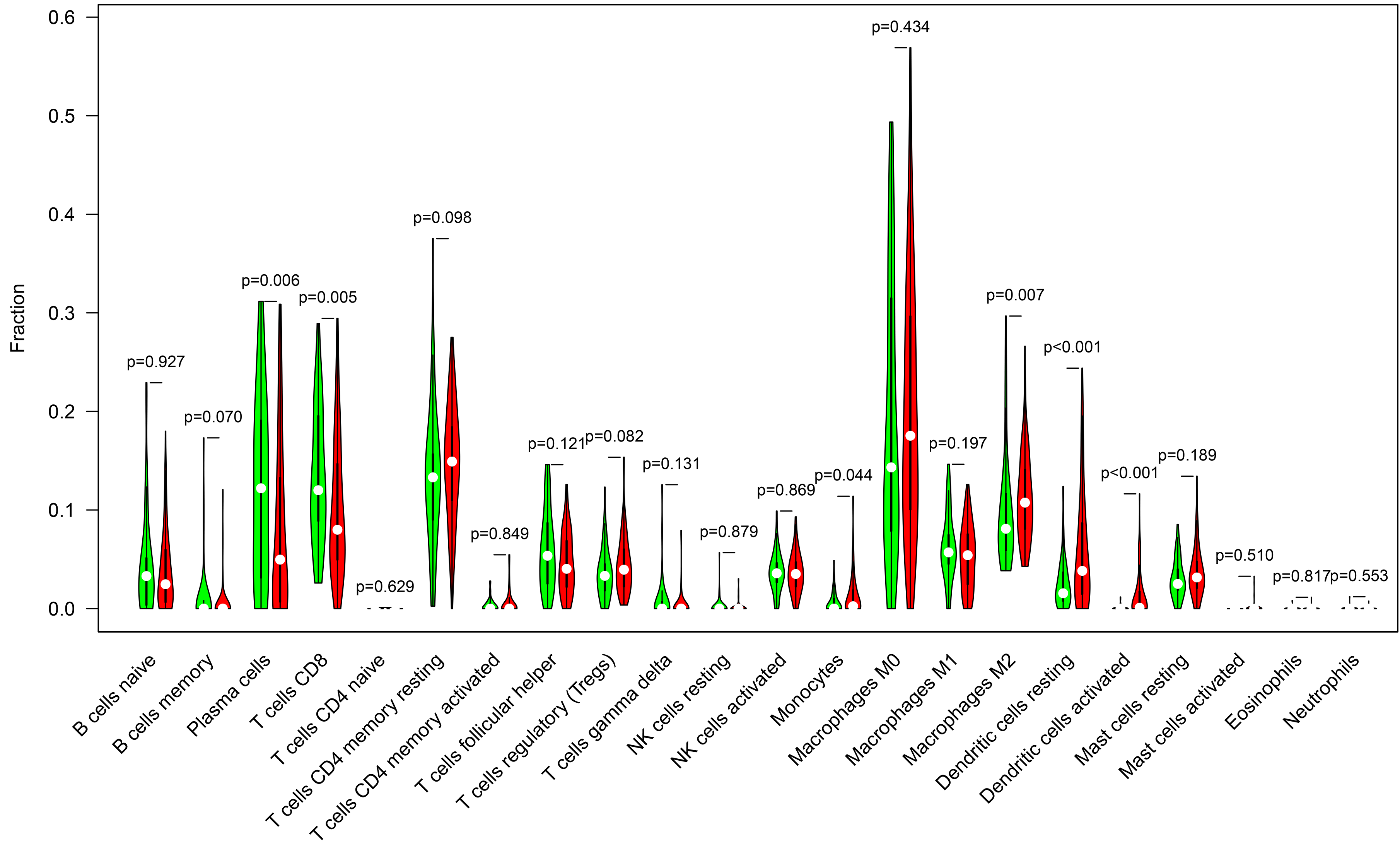


**Supplementary Figure 2** Difference analysis of immune cells in BC tumor samples. Wilcoxon rank sum was used for the significance test.

**Supplementary Figure 3**


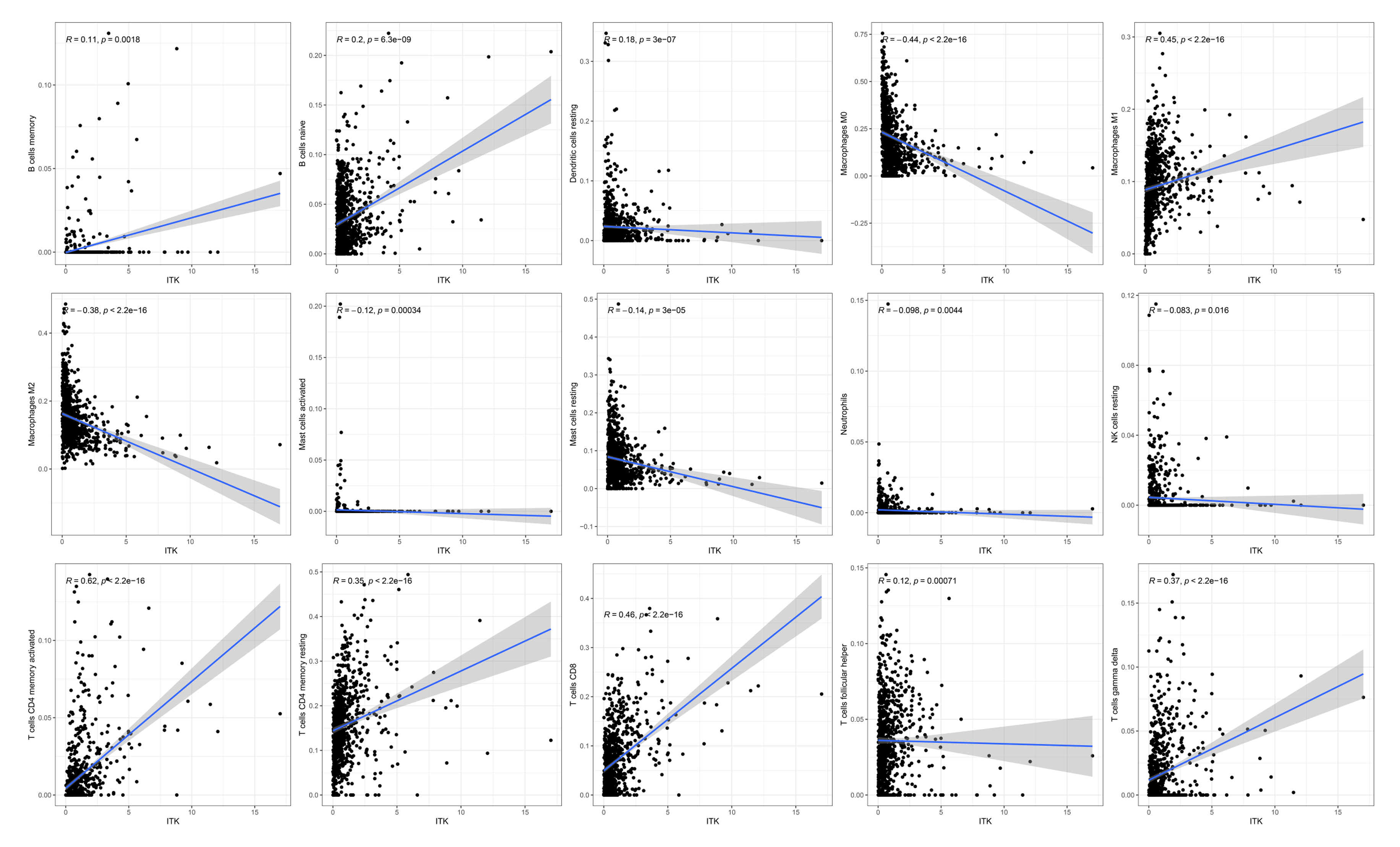


**Supplementary Figure 3** Correlation analysis of immune cells in BC tumor samples. Pearson coefficient was used for the correlation test.
